# Does a Nephron Deficit Exacerbate the Renal and Cardiovascular Effects of Obesity?

**DOI:** 10.1371/journal.pone.0073095

**Published:** 2013-09-03

**Authors:** Seshini Gurusinghe, Russell D. Brown, Xiaochu Cai, Chrishan S. Samuel, Sharon D. Ricardo, Merlin C. Thomas, Michelle M. Kett

**Affiliations:** 1 Department of Physiology, Monash University, Clayton, Victoria, Australia; 2 Department of Pharmacology, Clayton, Victoria, Australia; 3 Howard Florey Institute, University of Melbourne, Parkville, Victoria, Australia; 4 Department of Biochemistry and Molecular Biology, University of Melbourne, Parkville, Victoria, Australia; 5 Department of Anatomy and Cell Biology, Monash University, Clayton, Victoria, Australia; 6 Baker IDI Heart and Diabetes Institute, Prahran, Victoria, Australia; UCL Institute of Child Health, United Kingdom

## Abstract

It has been hypothesized that a reduced nephron endowment exacerbates the hypertensive and renal effects of obesity. We therefore examined the impact of diet-induced obesity on renal structure and function, and arterial pressure in a genetic model of reduced nephron endowment, the GDNF Heterozygous (HET) mouse. 6wk-old male GDNF WT and HET mice were placed on control or high fat (HFF) diet for 20 weeks. 24 hr arterial pressure, heart rate and activity (radiotelemetry), creatinine clearance and albumin excretion were measured, and kidneys collected (histopathology, collagen content). Bodyweights of HFF WT (50.6±1.2 g) and HET (48.8±1.4 g) mice were ∼14 g greater than control mice (37.3±1.3 g, 36.4±1.1 g respectively; Pdiet<0.001). Obesity led to significantly greater 24 hr MAP (Pdiet<0.001), heart rate (Pdiet<0.01) and lower locomotor activity (Pdiet<0.01) in HET and WT mice. Whilst there was no significant impact of genotype on 24 hr MAP response to obesity, night-time MAP of obese HET mice was significantly greater than obese WT mice (122.3±1.6 vs 116.9±1.3 mmHg; P<0.05). 24 hr creatinine clearance was 50%, and albumin excretion 180% greater in obese WT and HET mice compared to controls (Pdiet<0.05) but this response did not differ between genotypes. Obesity induced glomerulomegaly, glomerulosclerosis, tubulointerstitial expansion and increased collagen accumulation (total, collagen I, V and IV; Pdiet<0.001). Obese GDNF HET mice had exacerbated total renal collagen (P<0.01), and greater levels of the collagen I subtype compared to kidneys of obese WT mice. In summary, obese nephron-deficient GDNF HET mice were able to maintain the high creatinine clearances of obese WT mice but at the expense of higher MAP and greater renal fibrosis. Whilst modest, our findings support the hypothesis that a reduced nephron endowment increases the susceptibility to obesity-induced kidney disease and hypertension.

## Introduction

Obesity is associated with a wide spectrum of disorders including hypertension, vascular and cardiac dysfunction, and diabetes, leading to an increased risk of cardiovascular disease. Classically, kidney disease in obesity was considered secondary to cardiovascular risk factors, particularly hypertension and diabetes. However a high BMI has recently been demonstrated as an independent risk factor for chronic renal disease [1], [2]. The kidney undergoes significant hemodynamic and structural changes in obesity. In particular, the kidney demonstrates hyperfiltration (increased GFR and thus single nephron GFR; SNGFR) in line with the increased body mass [1]. In the long-term, obesity is associated with the appearance of kidney disease including albuminuria, glomerulosclerosis, mesangial matrix expansion, aberrant collagen deposition, lipid accumulation, inflammation, and a fall in GFR [1]–[3]. However not all individuals with obesity develop hypertension or chronic kidney disease. A hypothesis emerging in the literature is that a low nephron number increases the susceptibility to obesity-induced chronic kidney disease and hypertension through greater elevations in glomerular capillary pressure and exhaustion of renal functional reserve [1], [4]–[6]. Limited studies in humans with a nephron deficit (due to unilateral nephrectomy or unilateral renal agenesis) have suggested that BMI contributes to the progression of kidney disease in these individuals [1], [4]–[6]. However, the impact of obesity in the presence of a reduced nephron number has not been examined experimentally.

Glial cell line-derived neurotrophic factor (GDNF) is a crucial factor in the initiation of metanephric kidney development and the stimulation of nephrogenesis [7]–[9]. Homozygous null mutants are born without kidneys and die shortly after birth [7]–[9]. GDNF heterozygous mice are born with 2 small kidneys or a single kidney resulting in significant nephron deficits ranging from 30% for those with 2 kidneys to 65% for those with a single kidney [10]–[13]. Despite these deficits, GDNF heterozygous mice followed through to one year of age have normal arterial pressure and normal GFR due to significant increases in SNGFR [12]. We hypothesized that the combined effects of obesity and low nephron number driven increases in SNGFR would exhaust renal functional reserve and lead to exposure of the glomerulus to elevated systemic pressures, resulting in the initiation of renal disease and exacerbation of hypertension. Thus, this study aimed to examine the impact of obesity on arterial pressure and renal structure and function in a robust genetic model of low nephron number, the GDNF heterozygous (HET) mice, and in their wild-type (WT) littermates.

## Methods

Male GDNF HET and WT littermates were obtained from Monash Animal Services, Monash University. Mice were generated through the cross of female C57BL/6 mice with male GDNF HET mice. Genotype was determined by PCR using tail tissue taken from the mice at weaning and was confirmed post-mortem. Mice had *ad libitum* access to food and water and were housed in a room maintained at ∼25°C with a 12 hour light-dark cycle. All experiments were approved by the Monash University School of Biomedical Sciences Animal Ethics Committee and conducted in accordance with the Australian Code of Practice for the Care and Use of Animals for Scientific Purposes.

At 6 weeks of age GDNF WT and Het mice were housed individually and allocated to receive control diet (CONT; AIN93G from 6–12 weeks of age, Fat 7% w/w; 3.85 kCal/g: AIN93M from 12 weeks of age, Fat 4% w/w; 3.61 kCal/g, Specialty Feeds, Australia) or high fat diet (HFF; Fat 23.5% w/w; 4.54 kCal/g; SF04-001, Specialty Feeds, Australia). Mice continued to receive a control or high fat diet throughout the 20-week treatment period and subsequent experiments. Food intake and body weight of each mouse were recorded weekly.

### Renal Function and Arterial Pressure

After 20 weeks on control or high fat diet, mice were placed in metabolic cages and 24-hour urine samples obtained for assessment of renal electrolyte, osmolyte and albumin excretion, and creatinine concentration. Following the completion of renal function measurements, mice were anaesthetized (Isoflurane, 2.2–2.6% in 40% O_2_, 60% N_2_) and radiotelemetry probes (PA-C10, DSI, MN, USA) implanted in the carotid artery for the measurement of conscious arterial pressure [12], [14]. During surgery a blood sample (∼60 µl) was collected for measurement of haematocrit and plasma creatinine [12], [14]. Mice were given a 10 day recovery period following which arterial pressures, heart rate and locomotor activity values were recorded continuously for 7 days.

### Tissue Collection and Renal Histopathology

Following arterial pressure recording, mice were anesthetised (isoflurane) and a carotid arterial blood sample collected into chilled heparinized tubes for the measurement of plasma renin concentration (PRC; radioimmunassay [15]), free fatty acids (FFA), triacylglycerol (TAG), insulin and BUN. The kidneys were rapidly excised, decapsulated and weighed. The left kidney (cut into 4 transverse portions) was snap frozen in liquid nitrogen and stored at −70°C for analysis of collagen content. The right kidney was fixed in 4% paraformaldehyde, processed into paraffin, sectioned and stained with hematoxylin and eosin, periodic acid schiff and Masson’s Trichrome for blinded histological analysis or immunofluorescence (see below). At autopsy, 2 HET mice in the CONT and 2 HET mice in HFF groups were found to have a single kidney; these animals were removed from the analysis. The heart was excised and the left ventricle isolated and weighed.

### Renal Collagen Content

To determine total renal collagen content and concentration, frozen tissue was analyzed for hydroxyproline content as previously described [16], [17]. The values obtained for hydroxyproline were converted to collagen content by multiplying by a factor of 6.94 as hydroxyproline accounts for ∼14.4% of the amino acid composition of collagen [18]. These values were then expressed as a percentage of the kidney dry weight (collagen concentration). To quantify changes in interstitial collagen subtypes, SDS-PAGE analysis was performed [16], [17]. In brief, tissue samples underwent pepsin-digestion and the supernatant fluids run on 5% (w/v) acrylamide gels [16], [17]. The most prominent interstitial collagen subtypes present in the kidneys were types I and V. Densitometry was performed with OD values for each collagen subtype presented relative to CONT WT values [16], [17]. Collagen IV localization was determined by immunofluorescence microscopy [19]. In brief, a goat anti- human collagen type IV primary antibody (1∶50; Southern Biotech, Birmingham, AL) was used followed by a donkey anti-goat Alexa Fluor 555 (1∶500; Molecular Probes). All sections were counterstained with DAPI (1∶10,000; Molecular Probes), mounted with Fluorescent Mounting Medium (DakoCytomation) and analyzed using a Provis AX70 fluorescent microscope (Olympus, Tokyo, Japan), and F-view II digital camera (Soft Imaging System, Munster, Germany).

### Urine and Plasma Analysis

The concentration of urinary sodium (RAPIDChem 744, Siemens Healthcare Diagnostics Inc, Deerfield, IL, USA), osmolality (Advanced Osmometer 2020, Needham Heights, MA, USA) and albumin (Albuwell M; Exocell Inc., Philadelphia, PA, USA) were measured and 24 hr excretions calculated. Urinary and plasma creatinine concentration were determined using high performance liquid chromatography (HPLC) with creatinine clearance (Ccre) determined as an estimate of glomerular filtration rate (GFR) [12], [20]. Plasma FFA and TAG concentrations were measured by enzymatic colorimetric assays (FAs, Wako Pure Chemical Industries, Japan and GP-PAP reagent, Roche Diagnostic respectively), plasma insulin by ELISA (in house, Dept of Physiology, Monash University) and BUN was measured using iSTAT Chem8+ cartridges (Abbott, Australia).

### Statistical Analysis

Two-way ANOVA with the factors of genotype (Pgp) and diet (Pdiet) and interaction between those factors examined (Pint) and Bonferroni post-hoc analysis conducted where appropriate. Values are mean ± SEM.

## Results

### Diet Induced Obesity

**Figure 1:**
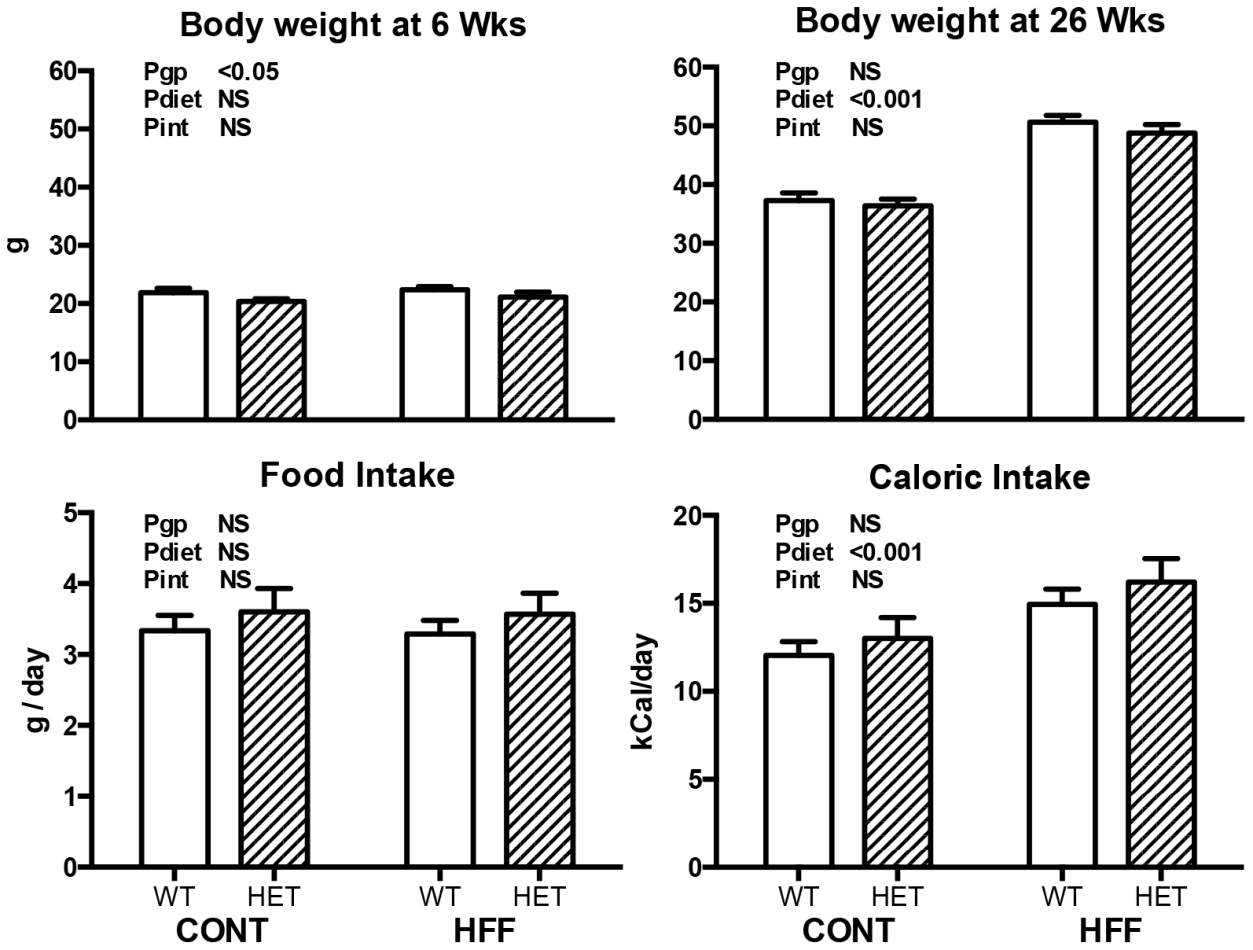
Body weight at commencement of the study (top left panel) and at the end of 20 week diet protocol (top right panel), and 24 hr food intake (bottom left panel) and 24 hr caloric intake (bottom right) in wildtype (WT; open bars) and GDNF HET (hatched bars) mice fed control (CONT) or high fat (HFF) diet. Data analyzed by two way ANOVA with the factors of genotype (gp), diet and interaction (int; diet x genotype). Values are mean ± SEM. n = WT-CONT 12, -HFF 13; HET-CONT 11, -HFF 8.

At 6 weeks of age mice were allocated to control or high fat diets. The bodyweights of HET mice at this age were slightly but significantly lower than WT mice but there were no significant differences between diet groups. After 20 weeks of diet treatment the genotype differences in bodyweight had disappeared and the body weights of HFF WT and HET mice were approximately 14 g greater that CONT WT and HET mice (Pdiet<0.001; Figure 1). There was no significant difference between the groups in food intake, though caloric intake was 25% higher in high fat fed obese mice (Pdiet<0.001; Figure 1).

### Arterial Pressure, Heart Rate & Activity

**Figure 2:**
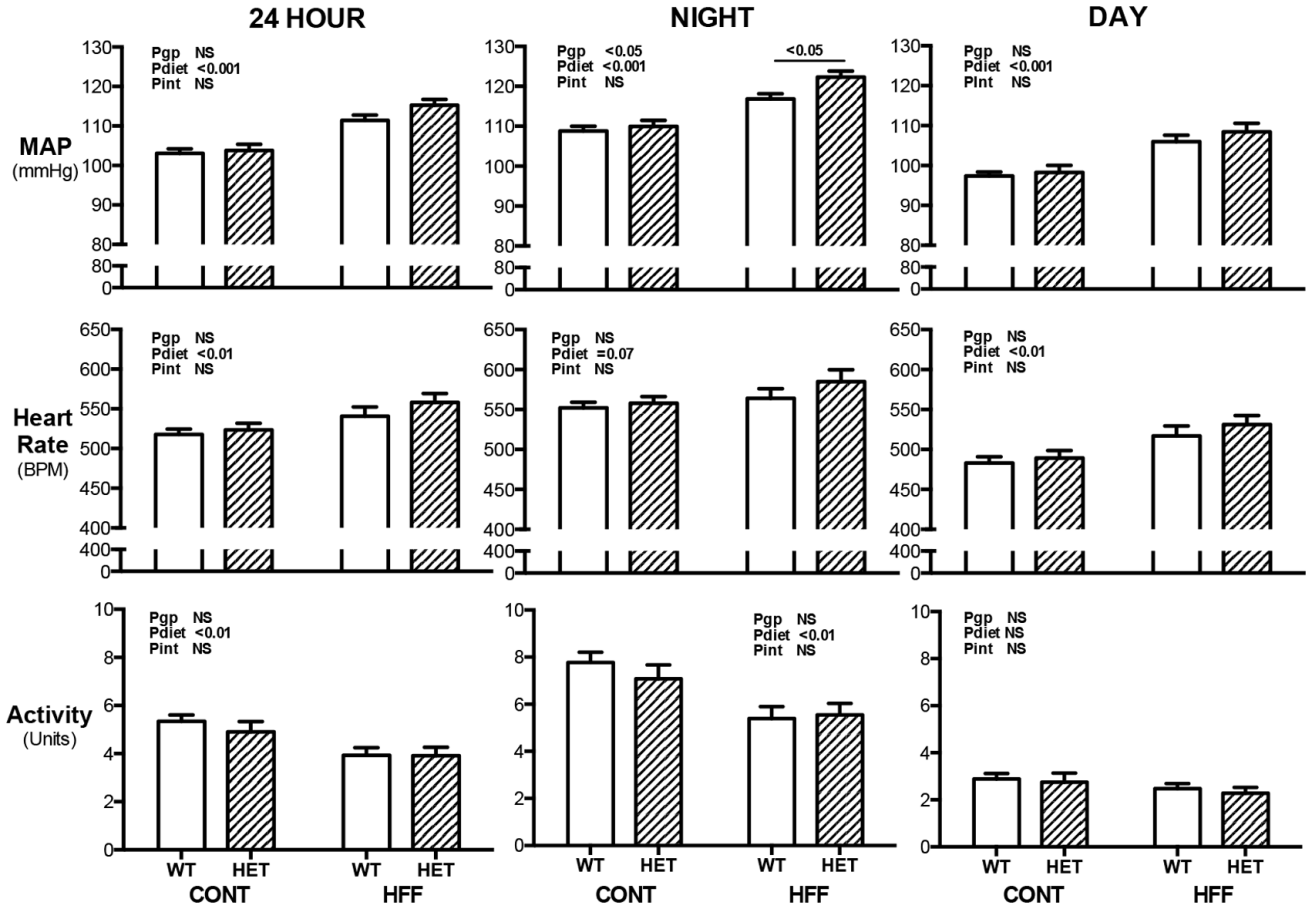
Mean arterial pressure (MAP; top panels), heart rate (middle panels) and locomotor activity (bottom panels) for 24hour period (left panels), night time (center) and day time (right panels) in wildtype (WT; open bars) and GDNF HET (hatched bars) mice fed control (CONT) or high fat (HFF) diet. Data analyzed by two way ANOVA with the factors of genotype (gp), diet and interaction (int; diet x genotype). *P<0.05 using Bonferonni post hoc analysis Values are mean ± SEM. n = WT-CONT 9, -HFF 8; HET-CONT 7, -HFF 5.

Diet-induced obesity led to significantly greater 24 hr, night- and day-time MAP in both WT and HET mice (Pdiet<0.001; Figure 2). The 24 hr- and day-time MAP responses to diet-induced obesity were not significantly different between the genotypes. Night-time MAP of HFF HET mice was modestly (5.4 mmHg), but significantly (P<0.05) greater than that of HFF WT mice (Figure 2).

Diet-induced obesity led to significant 24 hr tachycardia (Pdiet<0.01) that was present during the day- (Pdiet<0.01) but not night-time (Pdiet = 0.07). Diet induced obesity also led to a 20% reduction in 24 hr locomotor activity (Pdiet<0.01; Figure 2). This lower activity in obese mice was observed during night- (Pdiet<0.01; Figure 2) but not day-time. There were no significant differences in the HR or locomotor response of the genotypes to obesity.

### Renal Function

**Figure 3:**
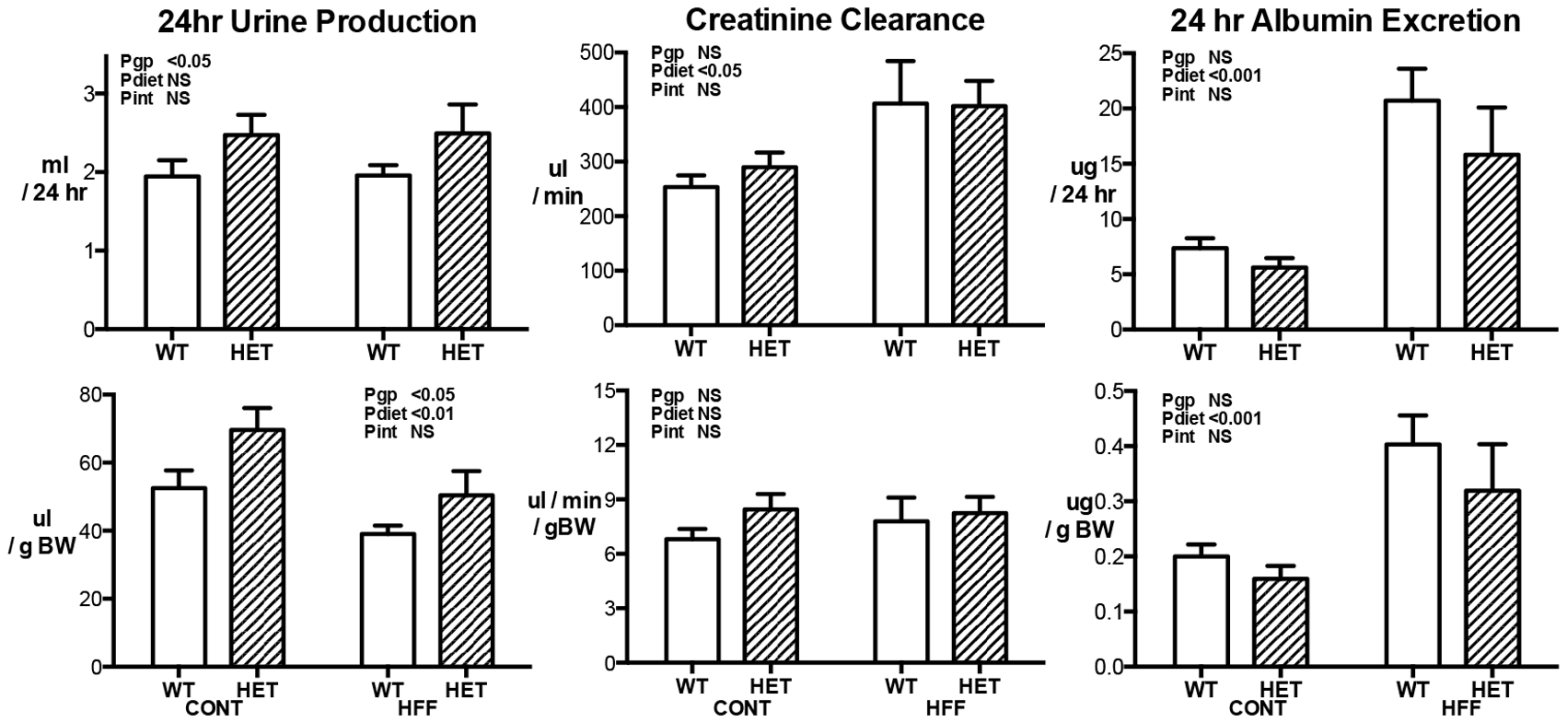
24hour urine production, creatinine clearance and 24 hr albumin excretion of wildtype (WT; open bars) and GDNF HET (hatched bars) mice fed control (CONT) or high fat (HFF) diet. Parameters are expressed in absolute (top panels) values and values adjusted for bodyweight (lower panels). Data analyzed by two way ANOVA with the factors of genotype (gp), diet and interaction (int; diet x genotype). Values are mean ± SEM. n = 7–13 per group.

Measures of 24 hr conscious renal function are presented in Figure 3 as both absolute values and values and corrected for bodyweight. Urine production in HET mice was significantly greater than WT mice (Pgp<0.05) in absolute terms and when adjusted for bodyweight (Pgp<0.05). Obesity did not impact on absolute urine production but resulted in a lower urine production per g bodyweight. There was no significant difference in the urine production response of the genotypes to diet-induced obesity. All differences in urine production were reflected in similar differences in water consumption (data not shown). There were no significant differences in creatinine clearance between WT and HET mice measured as either absolute or adjusted for bodyweight. Obese WT and HET mice had creatinine clearances that were over 50% greater than control mice (Pdiet<0.05; Figure 3), an affect that was similar between the two strains. When adjusted for body weights however there were no longer any significant differences in creatinine clearances across the 4 groups. 24hour albumin excretion was not significantly different between the genotypes but was 3-fold higher in obese WT and HET mice compared to control WT and HET mice (Pdiet<0.001; Figure 3). The higher albumin excretion in obese WT and HET mice persisted when values were adjusted for bodyweight (Pdiet<0.001). There was no significant difference in the albuminuria response of WT and HET mice to obesity. Albumin to creatinine ratio showed a similar pattern to albumin excretion (Data not shown).

Sodium excretion was also not significantly different amongst the four groups (CONT-WT 131.5±18.6, -HET 120.76±11.6; HFF-WT 108.1±9, HET 116.3±11.7 umol/day; n = 10, 9, 11, 6). Urine osmolality was significantly lower in HET mice compared to WT mice (Pgp<0.01; Data not shown), reflecting the higher urine output and water intake of HET mice, but there was no difference in the response of the genotypes to obesity.

### Terminal Tissue and Plasma Profile

At autopsy, there was no significant impact of genotype on bodyweights, however bodyweights of HFF WT and HET mice were approximately 35% greater than control WT and HET mice (Pdiet<0.001; Table 1). As expected, renal mass of HET mice was significantly less than WT mice (Pgp<0.001). Obese WT and HET mice had significantly greater renal mass than control mice but the increase in renal mass was not impacted on by genotype (Pdiet <0.01; Pint NS). Left ventricle weights were greater in WT compared to HET mice (Pgp<0.05), an effect that disappeared when corrected for bodyweight (Table 1). Left ventricle weights were significantly greater in obese WT and HET mice compared with control WT and HET mice (Pdiet<0.05; Table 1).

**Table 1:** Terminal Tissue and Plasma Profile.

	WT		HET		P		
	CONT	HFF	CONT	HFF	gp	diet	int
**MASS**							
**Body Weight**(g)	37.9±1.2	52.3±0.8	37.0±1.5	49.2±1.1	NS	<0.001	NS
**Kidney Wt**(mg)	341±14	403±16	262±18	284±14	<0.001	<0.05	NS
**Kid: Bwt**(mg/g)	9.02±0.33	7.72±0.32	7.22±0.66	5.77±0.26	<0.001	<0.001	NS
**LV**(mg)	106±4	114±3	94±4	99±4	<0.01	<0.05	NS
**LV: Bwt**(mg/g)	2.75±0.12	2.18±0.08	2.64±0.16	1.92±0.09	NS	<0.001	NS
**PLASMA**							
**Insulin**(pmol/L)	132±23	311±63	126±36	298±36	NS	<0.001	NS
**FFA**(mmol/L)	0.85±0.06	1.01±0.08	0.87±0.07	1.23±0.09	NS	<0.01	NS
**TAG**(mmol/L)	0.80±0.006	1.06±0.11	0.84±0.09	1.34±0.16	NS	<0.01	NS
**Cre**(umol/L)	12.9±1.5	10.4±1.7	10.5±1.1	9.29±1.0	NS	NS	NS
**BUN**(mmol/L)	6.8±0.7	9.5±0.9	8.3±0.6	11.5±0.5	NS	<0.001	NS

Terminal body weight, kidney weight, kidney to body weight ratio (Kid: Bwt), left ventricle (LV), left ventricle to body weight ratio (LV: Bwt) and non fasting plasma insulin, free fatty acid (FFA), triacylglycerol (TAG), blood urea nitrogen (BUN), and creatinine (Cre) in WT and GDNF HET mice fed control (CONT) or high fat (HFF) diet. Data analyzed by two way ANOVA with the factors of genotype (gp), diet and interaction (int; diet x genotype). Values are mean ± SEM. n = 6–11 per group.

WT and HET mice did not differ in plasma creatinine, nor was there a significant effect of obesity on plasma creatinine (Pgp, Pdiet NS; Table 1). BUN was not different between WT and HET mice however obese mice had significantly elevated BUN compared to control mice (Pdiet <0.001). Obese WT and HET mice had significantly elevated plasma insulin (Pdiet <0.001), FFA (Pdiet <0.01) and TAG (Pdiet <0.05). There were no significant differences between the genotypes for any of these parameters, nor were there any differences in the response of the genotypes to obesity for any of these parameters (Table 1).

### Renal Histology and Collagen Content

**Figure 4:**
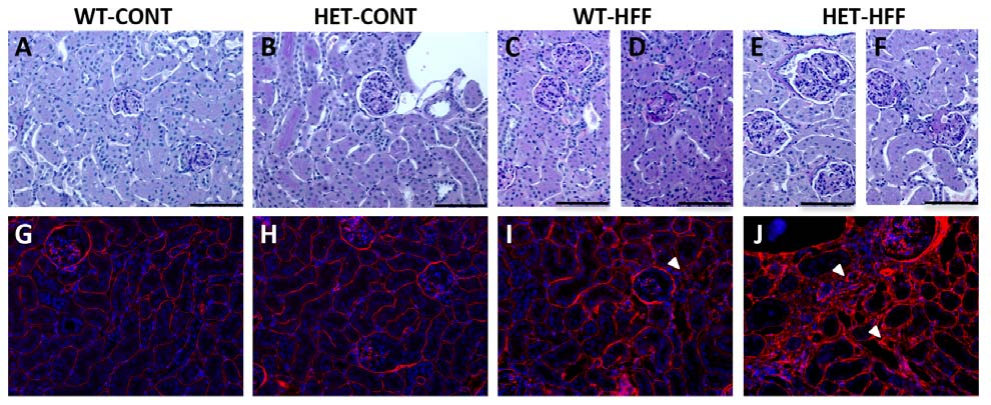
Typical histological features of kidneys of wildtype (WT) and GDNF HET mice fed control (CONT) or high fat (HFF) diet. **Panels A-F: High power images of typical glomeruli stained with periodic acid-Schiff (PAS).** Glomeruli of WT-CONT (A) and HET-CONT (B) appeared normal with no differences in PAS positive staining. Glomeruli of WT-HFF mice showed glomerular hypertrophy (C) and some presented with glomerulosclerosis (D). Kidneys of HET-HFF mice contained very large glomeruli (E) and glomeruli with glomerulosclerosis (F). Bar = 100 um. Panels G-J: Fluorescent micrographs of collagen IV (Red) immunostaining. Collagen IV immunostaining in control WT (G) and HET (H) kidneys presented as a fine matrix localized to basement membranes of renal tubules, Bowmans capsule and the intra and extraglomerular mesangium. In obese WT (I) and HET (J) kidneys, the accumulation of collagen IV protein was present and localized to the tubulointerstitium associated with expansion of the interstitial space (arrow). More extensive collagen IV was also evident in the glomerulointerstitium surrounding the glomerular Bowman’s capsule.

Renal histology of control HET mice was indistinguishable from control WT mice other than the presence of larger glomeruli in HET mice (Figure 4). Renal architecture of obese WT and HET mice was maintained, though they demonstrated expansion of the interstitial space and focal areas of inflammatory cell accumulation (not shown). Glomeruli of obese WT and HET mice appeared markedly larger with evidence of mesangial expansion and glomerulosclerosis (Figure 4).

**Figure 5:**
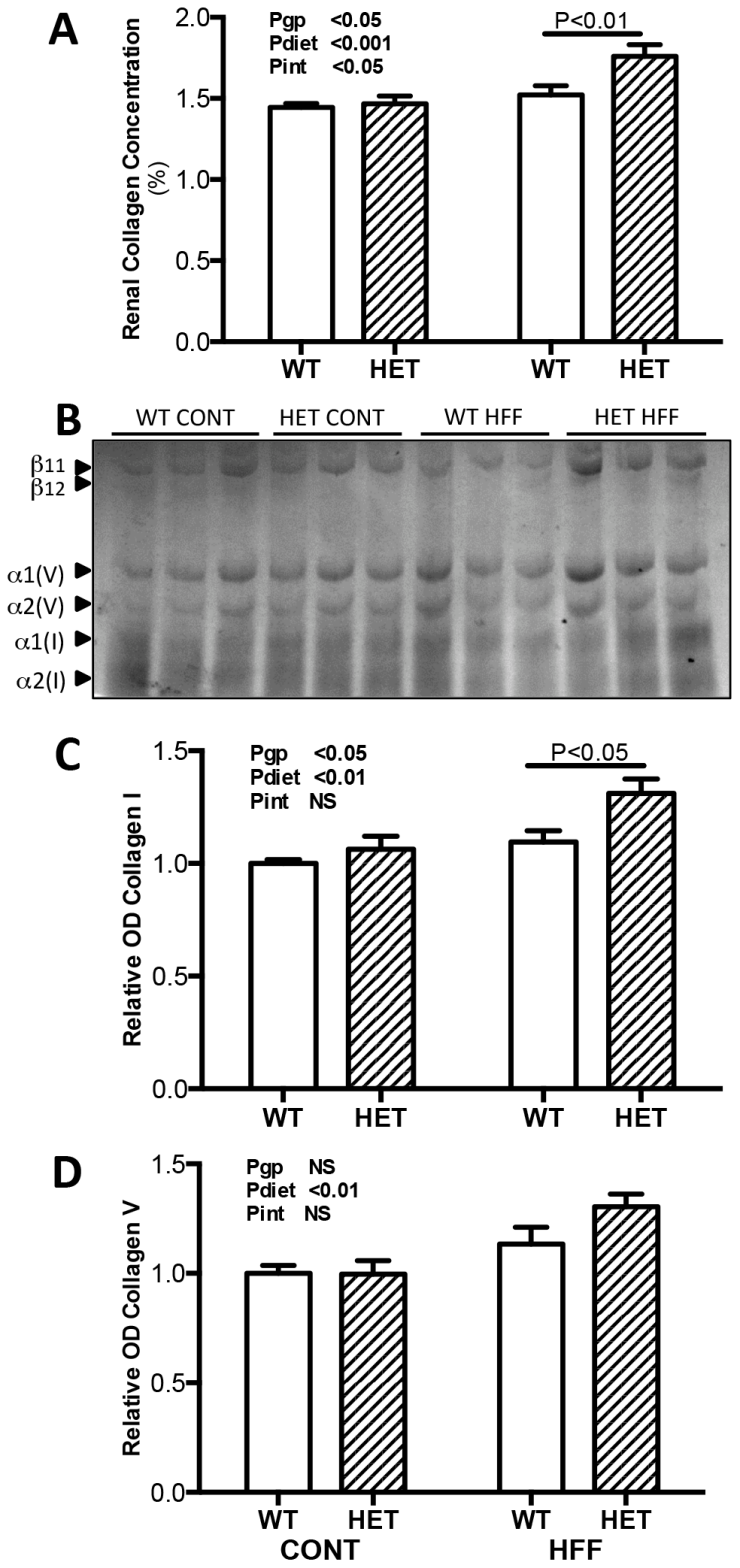
Renal Collagen Content. Panel A: Renal collagen concentration (collagen content expressed as percentage of dry tissue weight) of wildtype (WT; open bars; n = 8) and GDNF HET (hatched bars; n = 6) mice fed control (CONT) or high fat (HFF) diet. Panel B: SDS-PAGE acrylamide gel. Panel C and D: Densitometry of collagen I and collagen V subtypes with values expressed relative to control WT (n = 6/group). Data analyzed by two way ANOVA with the factors of genotype (gp), diet and interaction (int). *P<0.01 using Bonferonni post hoc analysis Values are mean ± SEM.

To quantify the degree of renal fibrosis in the 4 groups we measured total collagen concentration (% collagen content/dry weight tissue) by hydroxyproline assay and collagen subtypes by SDS-PAGE analysis. The localization of collagen IV was determined by immunofluorescence microscopy. Consistent with histological observations, we found no differences between collagen concentration of control WT and HET mice however obese WT and HET mice had significantly greater collagen levels than control mice (Pdiet <0.001; Figure 5). Further, the impact of obesity on collagen concentration was significantly greater in HET mice (Pint <0.05) such that the final collagen concentration of obese HET mice was almost 20% greater than obese WT (P<0.01; Figure 5). The changes in total collagen content were reflected by similar changes to collagen I and IV. The level of both subtypes were greater in obese compared to control WT and HET mice (Pdiet <0.01). Further the level of the collagen I subtype was significantly greater in obese HET mice compared to obese WT mice (P<0.05; Figure 5). Immunofluorescence labeling of collagen IV identified a fine matrix of collagen in control WT and HET kidneys that was localized to basement membranes of tubular and Bowman’s capsule epithelial cells and the intra and extra-glomerular mesangium (Figure 4). Kidneys of obese WT and HET mice demonstrated expanded interstitial space with marked increases in collagen IV accumulation in both tubulo-interstitial and glomerular- interstitial compartments (Figure 4).

## Discussion

Obesity and nephron deficiency each impact on single nephron hemodynamics increasing SNGFR, glomerular capillary pressure and tubular flow rates. The aims of the current study were to determine whether obesity, induced in a robust genetic model of nephron deficiency, would lead to renal insufficiency, renal disease and exacerbated hypertension. Twenty weeks of high fat feeding led to diet-induced obesity that was characterized by hyperinsulineamia, higher plasma FFA and TG, hypertension, renal hyperfiltration, albuminuria, renal injury and aberrant collagen deposition. Whole kidney hyperfiltration and albuminuria were not exacerbated in nephron deficient GDNF mice. However obese GDNF HET mice demonstrated a higher night-time MAP and greater renal collagen accumulation compared to obese WT mice. These results are consistent with the hypothesis that a nephron deficit increases the risk of cardiovascular and renal complications associated with obesity.

The GDNF heterozygous mouse is a genetic model of reduced nephron number that has been characterized by several groups, including ours, using unbiased stereological techniques [10]–[13], [21]. GDNF HET mice are born with either 2 small kidneys and a 30% nephron deficit or, in a more rare event, a single kidney and a 65% nephron deficit compared to WT littermates [10]–[13]. Glomeruli of GDNF HET mice undergo significant hypertrophy facilitated by increases in glomerular capillary length [21], mesangial and endothelial cell number and podocyte size [13]. The resulting increase in glomerular filtration surface area, combined with higher glomerular capillary pressure, is thought to contribute to the increased SNGFR of GDNF HET leading to normal whole kidney GFR and normal conscious arterial pressures [12], [13]. Despite the hyperfiltration and higher glomerular capillary pressures we, and others, have found no evidence of glomerulosclerosis or up-regulation of profibrotic genes in GDNF HET mice up to one year of age [12], [13]. Consistent with these previous findings, the current study found control GDNF HET mice had normal arterial pressures, plasma creatinine, creatinine clearance, albumin excretion, total collagen and levels of collagen subtypes I and V, and normal renal histology, other than the expected presence of larger glomeruli. Control GDNF HET mice also had a greater urine production than WT mice, again consistent with our previous reports [12].

Although, overwhelmingly, GDNF HET kidneys appear normal, Benz et al [13] did note that GDNF HET mice had a thickening of the glomerular basement membrane and a reduced density of podocytes surrounding glomerular capillaries. This led them to propose that these changes may predispose nephron deficient GDNF mice to glomerulosclerosis, glomerular injury and hypertension [13]. Like nephron deficiency, obesity is also associated with hyperfiltration and glomerular hypertension [1], [22], [23]. Thus, it has been hypothesized that the combined effects of obesity driven, and low nephron number driven increases in SNGFR may to lead to an exhaustion of the renal functional reserve, exposure of the glomerulus to elevated systemic pressures and the initiation of renal disease and exacerbation of hypertension [5], [24]. In this regard, a high BMI and obesity in humans have been linked to the appearance and progression of renal dysfunction and higher blood pressures following the removal of one kidney [4], [6], [25], [26]. Those living with one kidney also appear to demonstrate a reduced renal functional reserve [27], [28]. Further, Rook *et al* found that renal functional reserve of kidney donors was negatively correlated with increased BMI and absent in obese donors [28]. These findings have resulted in significant controversy of the use of obese individuals as living kidney donors [25], [29]. However the impact of obesity in an experiment model of nephron deficiency has not been examined.

C57Bl/6 mice, the background strain of the GDNF line, have been shown to be sensitive to the obesogenic effects of high fat feeding [3], [30]. High fat fed WT and GDNF HET mice gained double the weight of control fed mice over the 20 week feeding period and were 14 g heavier than control mice at the commencement of the study. Importantly, there was no difference in the obesogenic effects of high fat feeding between WT and HET mice with WT and HET mice showing similar increases in weight gain, plasma insulin, FFA and TG. Diet-induced obesity led to a 50% increase in creatinine clearance in WT and GDNF HET mice. The magnitude of this increase was in line with the increased body mass as when creatinine clearance was adjusted for bodyweight there was no longer a significant difference across the 4 groups. Further plasma creatinine levels were also not different across the groups. Despite normal plasma creatinine levels, obese WT and HET mice had elevated BUN compared to control mice, with no difference between genotypes. Whilst the elevated BUN of obese mice may suggest renal dysfunction, it is more likely due to the higher protein content of the high fat diet (22%) compared to the standard maintenance diet control mice were on (14%). We have previously reported that SNGFR of GDNF HET mice are between 2–4 fold greater than WT mice depending on the degree of nephron deficit. Given the known effects of obesity in the early phase to increase SNGFR, we had anticipated a reduced capacity of the kidneys of GDNF HET mice to undergo further hyperfiltration. However whole kidney creatinine clearance increased to similar levels in GDNF HET and WT mice suggesting that the nephrons of the GDNF HET mice were able to mount the appropriate physiological response to obesity. Thus, obese GDNF HET mice show no evidence of compromised renal functional reserve at the time point measured.

Obesity is associated with significant renal injury. Both obese GDNF HET and WT mice demonstrated the renal structural changes that are typical of the obese condition including glomerulomegaly, glomerulosclerosis, interstitial expansion, and inflammation [1], [3]. In order to obtain a quantitative measure of renal injury, in particular fibrosis and aberrant collagen deposition, we measured collagen concentration by hydroxyproline assay, collagen sub-type quantification by SDS-PAGE and localization by immunofluorescence. Total renal collagen concentration was not different between control WT and GDNF HET mice, but was significantly increased with obesity. This increase in renal collagen concentration with obesity was markedly greater in GDNF HET mice, approximately 25%, compared to WT mice where the increase in collagen concentration with obesity was only 8%. Whilst both collagen subtypes I and V were increased in obese mice, levels of collagen I were significantly greater in obese HET mice compared to obese WT mice suggesting that this subtype contributes to the greater total collagen content in obese HET mice. Immunofluorescence demonstrated that there was irregular collagen IV accumulation in the expanded tubule-interstitial and glomerular-interstitial spaces of obese HET and WT mice.

The mechanisms underlying the greater renal fibrosis in obese HET mice compared to WT mice are unclear but intra-tubular hydrodynamic forces may be a factor [31]. Rohatgi et al [31] have suggested that high tubular flow rates arising in states of high SNGFR, such as those occurring in obesity, contribute to interstitial fibrosis and inflammation via the activation of fluid shear stress regulated signaling processes. With 30% fewer nephrons, but similar creatinine clearance, obese HET mice will have significantly greater SNGFR and thus tubular flow rates compared to obese WT mice suggesting they are likely to have significantly greater tubular stretch and shear stress. Interestingly the effect of tubular shear stress on tubular interstitial fibrosis appears independent of hypertension and proteinuria [31].

Obesity led to significant albuminuria in GDNF HET and WT mice. Surprisingly, the greater renal fibrosis observed in obese GDNF HET mice was not reflected in greater albuminuria as detected by ELISA. The early phase of obesity is characterized by a state of volume expansion, renal hyperfiltration, renal and glomerular hypertrophy and the onset of injury in the form of glomerulosclerosis and tubulointerstitial fibrosis [22], [32], [33]. During this early phase of obesity, unaffected nephrons are believed to maintain the elevated GFR [22], [32], [33]. With maintained exposure to chronic obesity, the number of nephrons affected increases until it reaches a point where hyperfiltration can not be maintained and renal function declines leading to an increased risk of ESRD [2], [22], [32], [33]. We believe that after 20 weeks of high fat feeding, our obese mice are at this early phase of obesity. Further, our findings of exaggerated renal fibrosis in the obese HET mice suggest that they may demonstrate an accelerated decline in renal function with chronic obesity.

Using radiotelemetry, we found that obesity lead to significantly greater 24 hr MAP, higher heart rates, and lower locomotor activity compared to control mice. These changes, and the magnitude of these changes, are consistent with previous reports in diet-induced obesity models in mice, rats, rabbits and dogs [3], [34]–[37]. The increased heart rate and decreased locomotor activity of obese HET mice was similar to that of WT mice. Of note, the level of hypertension of obese HET mice was found to be significantly greater than obese WT mice. Whilst this exacerbated effect on arterial pressure did not reach significance for 24 hr MAP, it was evident during the night-time, or active phase of these nocturnal animals. Although the exacerbated hypertensive effect in GDNF HET mice was significant only for the night-time phase, as this is the awake or active phase of rodents, it has significant implications for cardiovascular risk. Longer-term studies will be required to determine if this exaggerated hypertensive response is exacerbated with longer duration of exposure to obesity.

Nephron deficiency (surgical and congenital) in animal models has been associated with increased MAP responses to short-term exposure to hypertensive stimuli such as high salt diets [12], [38]–[40]. Indeed we have previously shown that the magnitude of MAP responses of GDNF HET mice to 7 days of high salt diet was related to the degree of nephron deficit [12]. This is the first study to date to demonstrate an association between a nephron deficit and exaggerated hypertensive responses to diet-induced obesity. The mechanism underlying the modestly higher arterial pressure of GDNF HET mice remains unclear. At the time-point measured, after 20 weeks of high fat diet, there was no evidence of exhaustion of renal functional reserve or renal insufficiency that might underlie the greater hypertension as we had predicted. However, the greater collagen concentration and glomerular injury in obese GDNF HET mice suggest that vascular and/or tubular/interstitial injury may contribute.

There are large variations in nephron number in humans due to differences in the number we are born with, and differences in the rate of nephron loss through the course of our life due to surgery, disease or aging. The kidney has significant functional reserve to compensate for both congenital and acquired deficits in nephron number. We have shown however, that the impact of a secondary insult on renal reserve with obesity, leads to greater renal disease and higher arterial pressures in GDNF HET mice in the early phases of obesity. Our findings are thus supportive of the hypothesis that low nephron number increases the susceptibility to obesity-induced chronic kidney disease and hypertension.
